# Small Worldness in Dense and Weighted Connectomes

**DOI:** 10.3389/fphy.2016.00014

**Published:** 2016-05-10

**Authors:** Luis M. Colon-Perez, Michelle Couret, William Triplett, Catherine C. Price, Thomas H. Mareci

**Affiliations:** 1 Department of Psychiatry, University of Florida, Gainesville, FL, USA; 2 Department of Medicine, Columbia University in the City of New York, New York, NY, USA; 3 Department Biochemistry and Molecular Biology, University of Florida, Gainesville, FL, USA; 4 Department Clinical and Health Psychology, University of Florida, Gainesville, FL, USA

**Keywords:** weighted connectomes, small worldness, complex networks, tractography, brain topology

## Abstract

The human brain is a heterogeneous network of connected functional regions; however, most brain network studies assume that all brain connections can be described in a framework of binary connections. The brain is a complex structure of white matter tracts connected by a wide range of tract sizes, which suggests a broad range of connection strengths. Therefore, the assumption that the connections are binary yields an incomplete picture of the brain. Various thresholding methods have been used to remove spurious connections and reduce the graph density in binary networks. But these thresholds are arbitrary and make problematic the comparison of networks created at different thresholds. The heterogeneity of connection strengths can be represented in graph theory by applying weights to the network edges. Using our recently introduced edge weight parameter, we estimated the topological brain network organization using a complimentary weighted connectivity framework to the traditional framework of a binary network. To examine the reproducibility of brain networks in a controlled condition, we studied the topological network organization of a single healthy individual by acquiring 10 repeated diffusion-weighted magnetic resonance image datasets, over a 1-month period on the same scanner, and analyzing these networks with deterministic tractography. We applied a threshold to both the binary and weighted networks and determined that the extra degree of freedom that comes with the framework of weighting network connectivity provides a robust result as any threshold level. The proposed weighted connectivity framework provides a stable result and is able to demonstrate the small world property of brain networks in situations where the binary framework is inadequate and unable to demonstrate this network property.

## INTRODUCTION

The brain is a heterogeneous system comprised of a broad range of white matter (WM) connection strengths [1, 2]. Computational models of neuronal networks account for this heterogeneity by weighting the network edges. Chavez et al. found that weighted edges enhanced synchronization between network units [3]. To account for this enhanced synchronization and the broad range of large and small white matter tracts in the brain, the analysis of brain networks must include a connectivity weighting parameter to reflect the real underlying organizational structure of the brain [4, 5]. In addition to producing more realistic network models of the brain, weighting the edges adds an extra degree of freedom in calculated network parameters.

The topological organization of structural and functional brain networks has been extensively studied *in vivo* with magnetic resonance imaging (MRI) [6–10] and graph theory provides an appropriate framework in which to elucidate the topological organization of brain networks [4]. This approach has revealed several basic network characteristics of the brain, such as high clustering, short path lengths, modularity, and small world organization. In order to generate brain structural networks, white matter structural connections (network edges) are estimated from diffusion-weighting MRI (dMRI) using tractography between gray matter regions (network nodes). The resulting networks suggest only a small world organization for brain networks [11–14]. But these studies generally employ binary connectivity representations, which assume every connection between nodes is equivalent [11, 15], so the connection either exists or does not exist, because the strength of connection is not used to differentiate between connections. Weighted networks can differentiate the strength of connections, however weighted connectome studies have focused on the difference of specific metrics between healthy controls and pathological subjects. Weighted connectome studies have shown increased changes in node strength, efficiency, clustering between patients with various neurological diseases compared to healthy controls [16–20]. These studies illustrate that global changes may take place in the organization of network connections in neurological disorders. This suggests that the small-worldness topological index may also show these organizational changes in weighted connectomes.

Complex network models have been employed to describe various real world networks [21, 22] and weighted network parameters have been introduced, but a comprehensive weighted connectivity framework has not been reported which allows the estimation of brain network topological features, such as small worldness [23]. The usual binary framework approach to estimating the topology of brain networks includes the calculation of network metrics; degree distribution, path length and clustering coefficients [23]. In separate published works, all of these metrics have been generalized to their weighted counterparts. In the next section of this paper, these generalized metrics are reviewed then used to estimate the topological features (small worldness) of brain networks. This weighted network approach leads to a more realistic model of the brain network and a more robust characterization of the brain network topology.

## NETWORK METRICS

The adjacency matrix, *A*, is the most basic mathematical representation of network connectivity [24]. For simple and undirected graphs, the adjacency matrix is square and symmetric (i.e., *N* × *N*, where *N* is the number of nodes in the network):

(1) $$A=(\begin{array}{ccc} a_{11} & \cdots & a_{1n}\\ \vdots & \ddots & \vdots \\ a_{n1} & \cdots & a_{nn} \end{array}),$$

Unweighted networks, known as binary networks, are described in an adjacency matrix by either the presence or absence of an edge connecting any two nodes where these binary edges have the value,

(2) $$a_{ij}=\{\begin{array}{cc} 1, & \text{if an edge connects nodes }n_{i}\text{ and }n_{j}\\ 0, & \end{array}.$$

In a weighted networks, the adjacency matrix is modified by adding a weighting parameter to represent the connection strength of each edge,

(3) $$a_{ij}^{\prime }=a_{ij}\cdot w(e_{ij}).$$

Here the edge weight, *w*(*e_ij_*), characterizes the connectivity strength between each pair of nodes. The weighted adjacency matrix allows the calculation of network metrics, analogous to those derived from the binary adjacency matrix [25], and is the foundation of a framework to study the topology of weighted brain networks. Since translational diffusion is antipodal symmetric, only undirected graphs will be considered for dMRI-derived connections; hence, the elements of the binary and the weighted adjacency matrix will be symmetric (i.e., *a_ij_* = *a_ji_*). A simple overall measure of network connectivity, the graph density (ρ), is defined as the ratio of the number of edges in the network graph to the maximum number of possible edges. This parameter is used to quantify gross alterations in networks resulting from changes, such as a selected threshold.

### Node Connectivity

The node degree, *k_i_*, for the *i*^th^ node, *n_i_*, represents the number of nodes connected to node, *n_i_*, and is calculated from the binary adjacency matrix as follows,

(4) $$k_{i}=\sum_{j=1}^{N}a_{ij}.$$

A degree distribution, expressed as the probability that nodes have a degree, *k*, in the network of *N* nodes can be used to provide a simple representation of connectivity in the network. In weighted networks, a parameter analogous to degree (Equation 4) is the node connection strength, *s_i_* [26],

(5) $$s_{i}=\sum_{j=1}^{N}a_{ij}^{\prime }=\sum_{j=1}^{N}a_{ij}\cdot w(e_{ij}).$$

Like the degree distribution, the node connection strength distribution provides a simple representation of connectivity in a weighted network.

### Mean Geodesic Path Length

Another important network metric is the geodesic path length. Geodesic paths are useful because they represent efficient pathways for the transmission of information within the network. In binary networks, a geodesic path length, *d_ij_*, is the smallest number (dimensionless) of edges required to connect node *i* to node *j* [25]. The mean geodesic path length for a specific node in the brain has been associated with the efficiency of the overall network structure [27]. For an undirected binary network, the mean geodesic path length, *d̂_i_*, for node, *n_i_*, is defined as,

(6) $${\hat{d}}_{i}=\frac{\sum_{j=1,i\neq j}^{N}d_{ij}}{N-1}.$$

**FIGURE 1:**
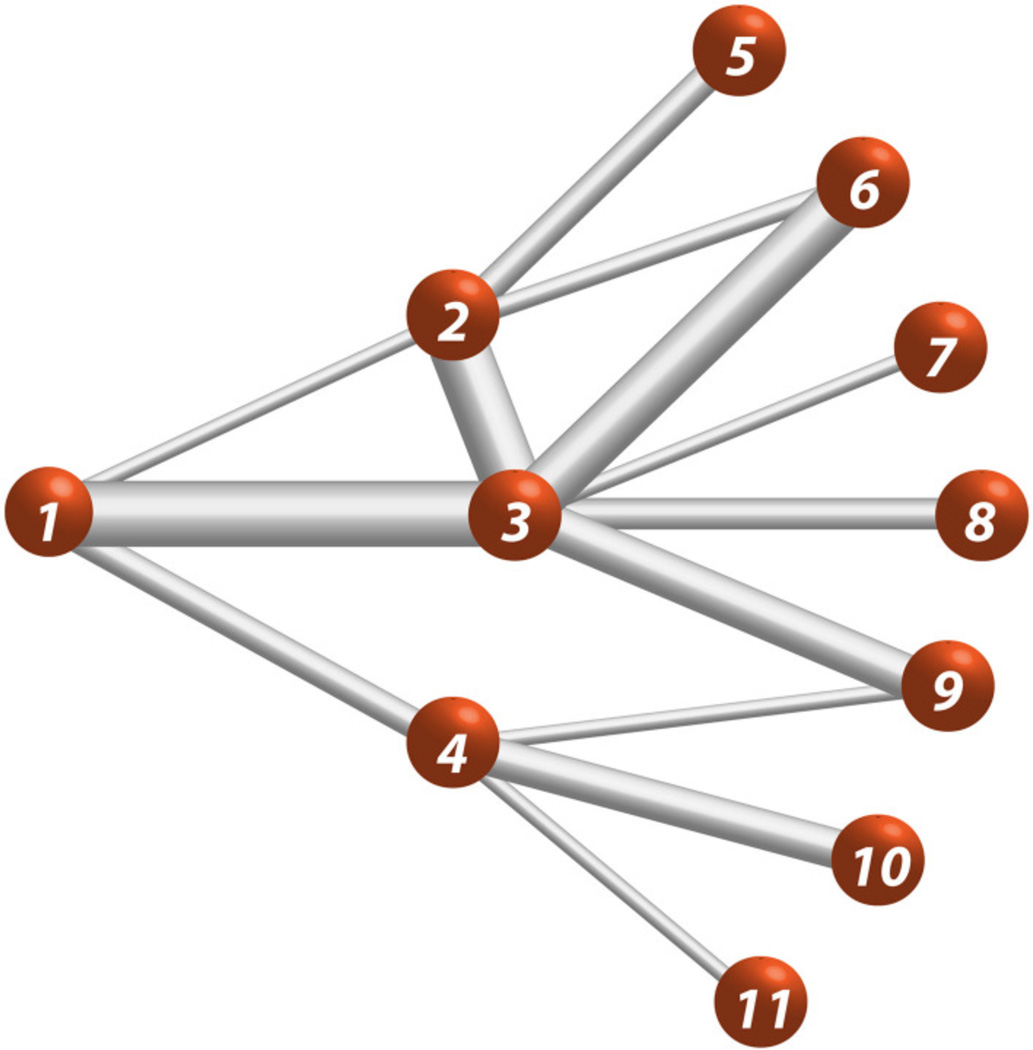
Eleven-node weighted network Edge thickness represents the relative edge weight strength, e.g., *e*_13_ > *e*_14_.

Starting from the definition of the binary geodesic path length, a geodesic path length for weighted networks, $d_{ij}^{w}$, can be defined using a Dijkstra’s algorithm [28]: first define the paths with the same and smallest number of edges between nodes *n_i_* and *n_j_*, then choose the path with highest sum of edge weights. An illustration of the algorithm is shown in the graph of Figure 1. Using the weighted adjacency matrix, the algorithm starts by determining the low-cost path between nodes connected by one edge. For the path between nodes *n*_1_ and *n*_2_, the original form of Dijkstra’s algorithm would assign the lowest-cost (highest connection strength) to the path along the edges from *n*_1_ to *n*_3_, and *n*_3_ to *n*_2_ because the total connection strength is stronger than the direct edge between node *n*_1_ and *n*_2_. For brain networks, Dijkstra’s algorithm is modified to give highest priority to the connections with the fewest number of edges then sort those to find the lowest cost. Therefore, the low-cost path between nodes *n*_1_ and *n*_2_ is the direct edge between node*n*_1_ and *n*_2_. As the modified Dijkstra’s algorithm continues, the low-cost path is determined for nodes connected by two or more edges. For example, the low-cost path from node *n*_1_ to *n*_6_ is found by starting at node *n*_1_, which has three neighboring nodes, and two of these nodes have a two-edge path, which connect to *n*_6_. The algorithm compares $d_{16}^{w}$ for the path *w*(*e*_12_) + *w*(*e*_26_) and *w*(*e*_13_) + *w*(*e*_36_). With the edge weight represented by the thickness of the edge, a visual inspection of each these paths in Figure 1 shows that *w*(*e*_12_) + *w*(*e*_26_) < *w*(*e*_13_) + *w*(*e*_36_); therefore in this example, the low-cost path is $d_{16}^{w}=w(e_{13})+w(e_{36})$. In general, the process of determining the low-cost paths starting at node *n_i_* begins with directly connected nodes and is repeated for path lengths with increasing number of steps until all of the (*N* −1) low-cost paths are determined. This results in the following form of the mean (low-cost) geodesic weighted-path length for node *i*;

(7) $${\hat{d}}_{i}^{w}=\frac{\sum_{\left(j=1,i\neq j\right)}^{N}d_{ij}^{w}}{N-1}.$$

In the context of brain networks, a step along the mean geodesic weighted-path is associated with the strongest connection (i.e., highest sum of edge weights) between nodes.

### Clustering Coefficient

The motifs of connections within the network provide information about network structure and how the network functions. The clustering coefficient, *c_i_*, is a measure of the motif of triangular connections for the *n_i_* node, which quantifies the local connectivity around this node by measuring the connectivity between the neighboring nodes directly connected to node *i*. The clustering coefficient in binary form is given by [23, 24],

(8) $$c_{i}=\frac{2E_{jm}}{k_{i}(k_{i}-1)}=\frac{2}{k_{i}(k_{i}-1)}[\frac{1}{2}\sum_{j,m=1}^{N}a_{ij}a_{jm}a_{mi}]\;=\frac{1}{k_{i}(k_{i}-1)}\sum_{j,m=1}^{N}a_{ij}a_{jm}a_{mi},$$

where *E_jm_* (defined in square brackets) is the number of edges connecting the neighbors of node *i* (i.e., the number of connected closed triangles, where the three nodes involving node *i* are fully connected). The normalization *k_i_*(*k_i_*− 1)/2 represents the number of possible connections among the neighbors of node *i*. In the brain, the clustering coefficient has been associated with specialized processing (e.g., sensory input analysis, such as visual and auditory) [27] where nearby nodes work together to achieve complex tasks. Therefore, high clustering of the neighboring nodes ultimately allows efficient communication and complex task processing.

Weighted network generalizations of the binary clustering coefficient in Equation (8) have been reviewed by Saramaki et al. [29], who conclude that none of the proposed generalizations provide an all-purpose weighted clustering coefficient, and they suggest the characterization of a network using a clustering coefficient should be made from two perspectives: binary and weighted. Onnela et al. [30] introduced a weighted clustering coefficient, *c_i,O_* in Equation (9) below, that replaces the sum of triangular binary adjacency coefficients with the scaled edge weights (scaled by the maximum edge weight in the network, as shown in Equation 11 below).

(9) $$c_{i,O}=\frac{1}{k_{i}(k_{i}-1)}\sum_{j,m=1}^{N}{\left[ŵ(e_{ij})ŵ(e_{jm})ŵ(e_{mi})\right]}^{1/3},$$

Also Zhang and Horvarth [31], introduced a weighted clustering coefficient, *c_i,Z_* in Equation (10) below, using the sum over the product of each triangle of normalized edge weights in the numerator, but included a normalization factor in the dominator using scaled edge weights.

(10) $$c_{i,Z}=\frac{\sum_{j,m=1}^{N}ŵ(e_{ij})ŵ(e_{jm})ŵ(e_{mi})}{{\left(\sum_{j=1}^{N}ŵ(e_{ij})\right)}^{2}-\sum_{j=1}^{N}ŵ{\left(e_{ij}\right)}^{2}},$$

where

(11) $$ŵ(e_{ij})=\frac{w(e_{ij})}{\text{max}(w(e))}.$$

As *ŵ*(*e_ij_*) → 1 (or *w*(*e_ij_*) → 1) in all triangles, both of these weighted clustering coefficients approach *c_i_*, thus ensuring that the results in weighted networks will yield the expected binary results when the weighted edges are converted to binary edges. In this study, these weighted clustering coefficients will be compared to the binary clustering coefficient, as suggested by Saramaki et al. [29].

### Small Worldness

Many real-world networks have been shown to have high overall clustering and short average path lengths, which reflect regional specialization and information transfer efficiency [32], and are classified as small world networks. Small world networks are more clustered than random networks, defined with the Erdös’ and Rényi’s model of random graphs [33], yet display similar geodesic path lengths. Humphries and Gurney [34] introduced a small-worldness metric which uses the networkaverage clustering coefficient and path length values, relative to these metrics for random networks, to provide an overview of connectivity in the entire network. The small-worldness metric is obtained by taking the ratio of two ratios: (1) the ratio of the average clustering coefficient (Equations 8–10) of the brain network, *c_g_*, to clustering coefficient, *c_N_*, of a random networks,

(12) $$\gamma =\frac{c_{g}}{c_{N}},$$

and (Equation 2) the ratio of average geodesic path length [average of all nodes for the path of Equations (6 and 7)] of brain networks, *l_g_*, to the average geodesic path length of a random networks, *l_N_*,

(13) $$\lambda =\frac{l_{g}}{l_{N}}.$$

Then the small-worldness metric, *sw*, is given by the ratio of Equations (12 and 13),

(14) $$sw=\frac{\gamma }{\lambda }.$$

In a binary framework, small world networks have γ > 1 and λ ~ 1 and present a high degree of connections among the neighbors of any node, compared to a random network, but small average path lengths are preserved. In weighted networks, any node may present a strong level of connectivity among its neighbors, while preserving small path lengths, so that the small-worldness metric can be used to characterize weighted networks.

## MATERIALS AND METHODS

### MRI Acquisition

The University of Florida Institutional Review Board approved this human study. One healthy subject was scanned 10 times over the course of 1 month, which provided 10 controlled brain data set from which to determine the network properties across different MRI acquisitions [35, 36]. In all 10 sessions the subject was awake and scans were acquired roughly at the same time of the day (~7:00 a.m.). The subject was scanned in a 3 T Siemens Verio system in the Shands Hospital of the University of Florida [37]. High angular resolution diffusion imaging (HARDI) data was obtained with a spin echo preparation and an echo planar imaging [38] readout using the following parameters: TR/TE = 17300/81 ms, two scans without diffusion weighting, six diffusion gradient directions with *b*-value of 100 s/mm^2^, and 64 diffusion gradient directions with *b*-value of 1000 s/mm^2^. The diffusion gradients were distributed following a scheme of electrostatic repulsion [39]. The diffusion-weighted images covered the entire brain with an isotropic resolution of 2.0 mm, field of view (FOV) of 256 × 256 mm and 73 slices. To increase the spatial resolution, data were interpolated with cubic convolution [40] using the CONGRID function in the Interactive Data Language (IDL; Exelis Visual Information Systems, McLean, VA) to a voxel dimension of 1 mm^3^. In addition, a high-resolution T1 structural scan of the entire brain was acquired with TR/TE = 2500/3.77 ms, resolution 1 mm isotropic, FOV of 256 × 256 mm and 176 slices.

### Post Processing

The diffusion weighted scans were corrected for eddy current distortion using FSL’s *eddy_correct* algorithm [41]. FA and AD maps were created with in-house software using IDL. The displacement probability function (DPF) was calculated for each voxel using a mixture of Wishart distribution (MOW) [42] implemented with in-house, C-based software. The MOW distributions were obtained using a distribution of positive definite matrices (rank-2 tensors) that allow the estimation of multiple fiber orientations in each MR voxel. Then the fiber orientations in each voxel are identified as the DPF maxima with probability of 0.5 or larger.

Using the MOW fiber orientations, deterministic tractography was performed to generate streamline maps throughout the entire brain. Tractography was performed following a modified version of the fiber assignment by continuous tracking (FACT) algorithm [43], in which the direction with the least angular deviation along the incoming fiber path was selected to be continued at each iteration of the tractography process. Fiber maps were calculated using an in-house, C-based software. Seed points were placed uniformly throughout the MR voxels to avoid the ambiguity of random placement of seed points. From each seed point, one streamline is launched bi-directionally for each estimated displacement probability maxima contained in the voxel. Each streamline is propagated by stepping half of the MR voxel width in the direction that is most in line with the streamline’s current direction of propagation. Tractography was performed with 125 seeds per voxel, a fiber step size of 0.5 of the voxel size, using the following criteria: no step-to-step track deviations greater than 50°, and no tracking into voxels with low anisotropy values (FA < 0.05). If the estimated track does not meet these criteria, the streamline is stopped.

### Network Construction

Brain networks were generated from 68 anatomical nodes segmented [44, 45] using the structural T1-weighted image with an automatic segmentation algorithm in FreeSurfer (Laboratory for Computational Neuroimaging, A. A. Martinos Center for Biomedical Imaging, Charlestown, MA). The structural image and diffusion weighted images was spatially registered using FSL’s FLIRT [46] algorithm, using an affine transformation. Employing FLIRT’s transformation matrix output, the FreeSurfer derived nodes were then registered to DWI using FSL’s ApplyXFM.

The network edges are defined by streamlines connecting any two nodes and the edge weight is defined as [37],

(15) $$w(e_{ij})=(\frac{V_{\text{voxel}}}{P_{\text{voxel}}})(\frac{2}{A_{i}+A_{j}})\sum_{p=1}^{P_{\text{voxel}}}\sum_{m=1}^{M}\frac{\chi_{R}(f_{p,m})}{l(f_{p,m})},$$

where

(16) $$\chi_{R}(f_{p,m})=\{\begin{array}{c} 1,f_{p,m}\in R\\ 0,f_{p,m}\notin R \end{array},$$

*V_voxel_* is the MR voxel volume, *P_voxel_* is the number of seed points per voxel,*A* is the surface area of each node, *M* is the number of voxels making up the edge, *f_p,m_* is the streamline originating from seed point *p* in voxel *m*, *l*(*f_p,m_*) is length of *f_p,m_* streamline making up the edge, and χ_*R*_(*f_p,m_*), is the characteristic function for the set *R* of streamlines connecting nodes *n_i_* and *n_j_*. The characteristic function removes (i.e., filters) streamlines that originate within the nodes and voxels that do not represent the WM path connecting the nodes [37]. This form of the edge weight calculation eliminates the effect of length of the streamlines, seeding paradigm, and tractography-specific experimental factors from the calculation [37]. The inverse sum along with the characteristic function (Equation 16) yields the number of streamline fibers connecting the nodes, and the normalization by the average surface area of the nodes reduces the bias introduced by node size. This form of the edge weight allows the quantification of white matter connection strength between any two nodes in a dimensionless and scale invariant manner.

In brain networks created from tractography, thresholds are usually employed to reduce the number of false edges and the effects of voxel volume averaging in network measures. In binary networks, thresholding to produce a sparse networks (i.e., the number of edges is much less than 30% of the total number of possible edges) is used to estimate better the topological properties of binary networks, since dense networks are highly connected and may lead to an almost complete graph (every node connected to all other nodes in the graph). But thresholding binary networks introduce two problems in networks calculations. Firstly the threshold selection is arbitrary and secondly networks with different thresholds are not independent samples [47]. Arbitrarily chosen thresholds do not ensure that small and real edges are included or that artifactual edges are completely eliminated, since there is no clear criterion to achieve the elimination of artifactual streamlines. Also, the statistical results obtained for all binary networks will depend on the selected thresholds, since binary networks created at different thresholds are pseudo replicas of group-level networks [47]. This means that the network results obtained from the same data, expressed as a sparse network, are also obtained in a dense network, but not vice-versa. This indicates that there is direct dependence of the calculated network properties on the applied threshold, which leads to different results at different thresholds. In this paper, a threshold parameter is selected that can be applied to both binary and weighted networks. Then network metrics (discussed in Section Network Metrics) are calculated at different threshold levels in order to determine whether weighted networks allow better estimation of an appropriate threshold or if it is possible to eliminate thresholds from the analysis.

In this work we will use the streamline fiber count is a threshold parameter. For the weighted network discussed here, a high seed density (*P_voxel_* = 125) is used to maximize the accuracy of the calculated edge weights [37]. But weighted-network thresholds should be set low enough so that “weak” edges are not removed just to create sparse networks. To illustrate how the streamline-fiber-count threshold approach can be used, assume that the “smallest” edge that will contribute to the brain network has a length of 2 mm and streamline fibers that are entirely contained within two voxels. Using parameters from Equation (15), a maximum of 250 streamlines (*M* × *P* = 2 × 125 = 250) should be connecting the nodes. Therefore, a threshold of 25 streamlines would retain all edges that have at least ~10% (i.e., 25/250) of the streamlines that can potentially make this “smallest” edge. Therefore, using the number of streamlines connecting any two nodes as a threshold parameter, and not the edge weight value, will allow this threshold to be applied to edges of both the binary and weighted brain network. Considering the high volume of seeds-per-voxel used in this study, four threshold values will be used to examine the effects of thresholding on calculated binary and weighted network parameters. First the network metrics will be calculated without applying any threshold, i.e., any two nodes connected by at least one streamline will have an edge, then edges that contain at least 25, 50, and 125 or more streamlines will be considered valid edges.

All network metrics described in the previous section are calculated with in-house code written in R (http://cran.us.r-project.org/) and the network package in R (http://cran.r-project.org/web/packages/network/index.html) was used to create and modify networks, and create relational data within the R interface. Three dimensional brain networks were displayed with BrainNet [48].

### Null Hypothesis Graphs

Network metrics are influenced by the decisions made when the network is generated (e.g., identification of nodes and edges). Therefore, to test the significance of differences in the metrics (i.e., clustering coefficients and path lengths) between binary and weighted brain networks, these metrics are compared to the same metrics obtained for a null hypothesis network [49]. A null-hypothesis binary-network is formed by assigning edges at random using two constraints: (1) the number of nodes and (2) the degree distribution remains identical to the original brain network. Since both the brain network and null hypothesis network are constrained by the number of nodes and the degree distribution, differences between them are not due to local differences in connectivity (i.e., node degree discrepancies) [7].

To create weighted null hypothesis networks, a third constraint is used to account for the additional degree of freedom resulting from weighting the edges. This third constraint requires that the edge weight distributions in the null hypothesis and original brain network are the same [14]. The process of creating the null hypothesis network starts by storing lists of the edge weight values and the node degree distribution (binary) for each brain network [49]. Then starting with the same number of nodes as the brain network, the edges are randomly connected in node pairs, while preserving the node degree distribution. As each edge is assigned, an edge weight is randomly selected from the edge weight list to create the null hypothesis network. Ultimately, this creates a random network with the same node degree distribution (i.e., binary null hypothesis network) and edge weight distribution (i.e., weighted null hypothesis network) as the calculated brain network.

## RESULTS

**FIGURE 2:**
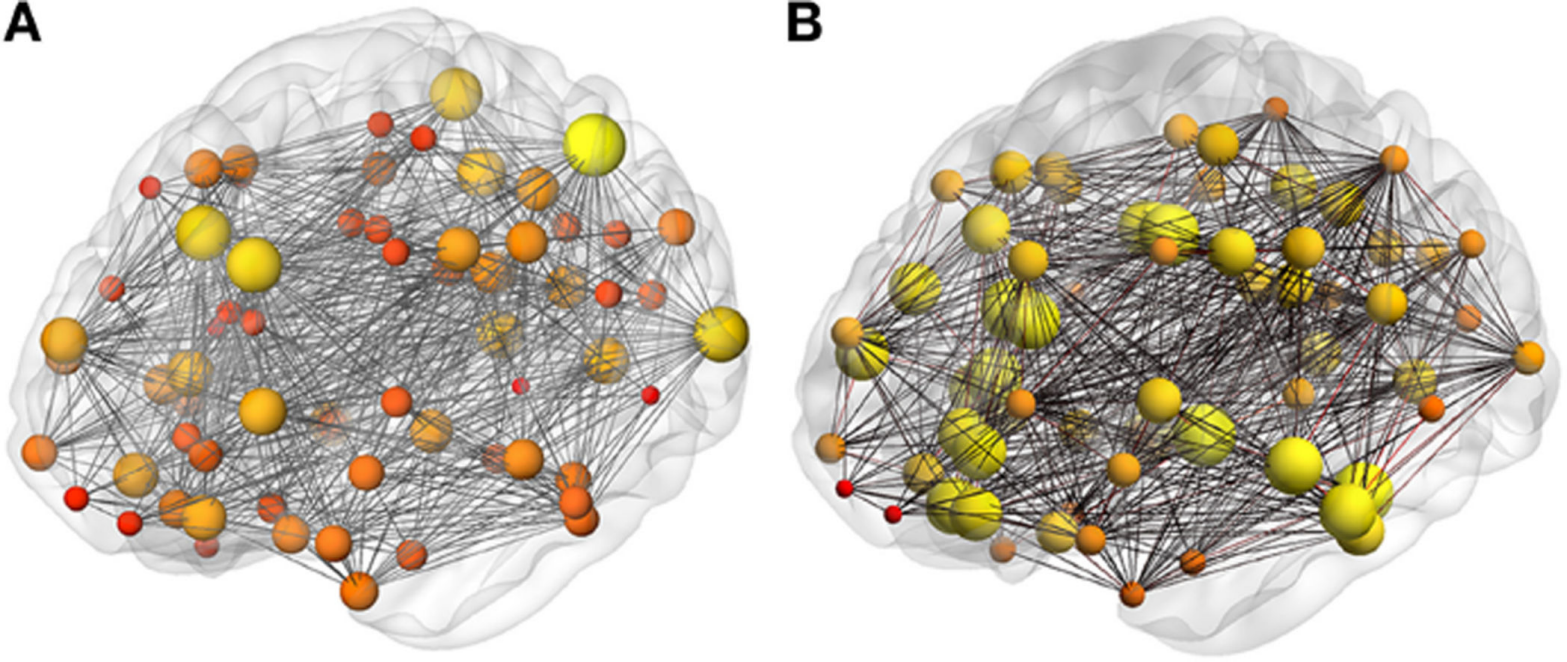
Brain networks Representative **(A)** binary and **(B)** weighted network. The nodes in the binary network are scaled by the node degree and in the weighted network by the connection node strength.

**FIGURE 3:**
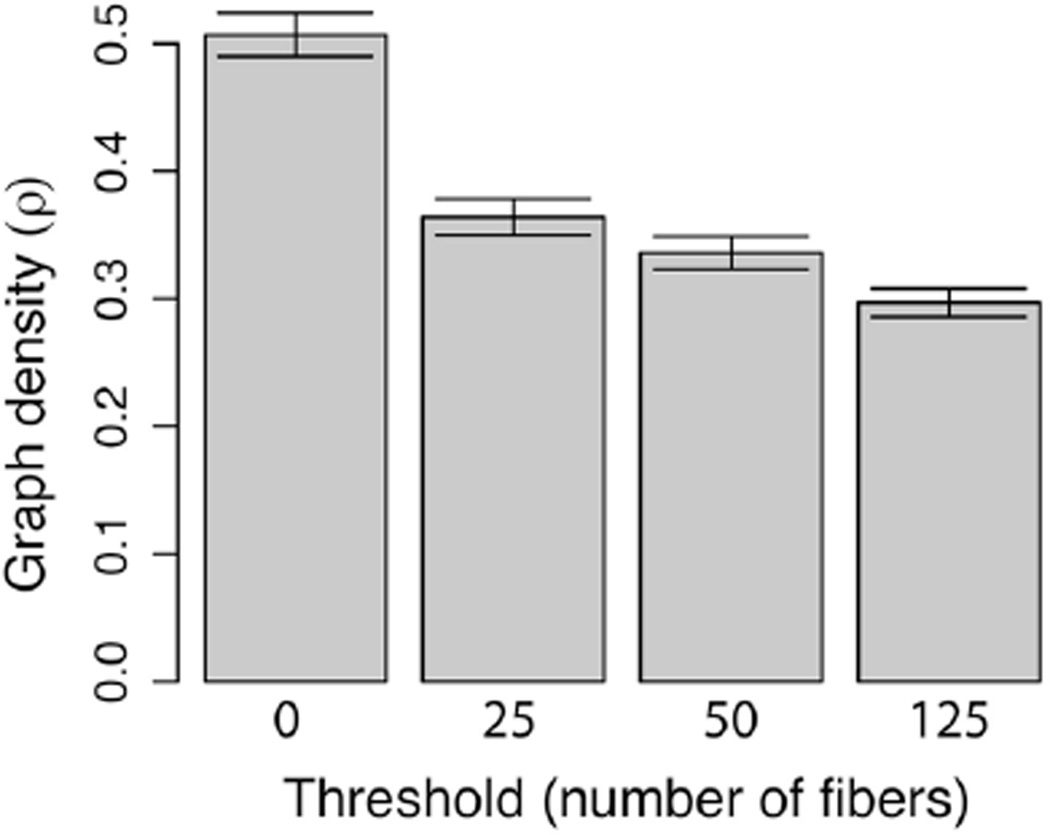
Graph density vs. threshold Density is obtained by taking the ratio of the number of edges in a graph to the total possible number of edges in the graph. The threshold is the number of steamline fibers below which an edge is removed from the network.

Both binary and weighted brain networks are shown in Figure 2 at the threshold of 125 or more fibers. The mean binary graph density at different thresholds for all 10 networks, derived from the 10 dMRI measurements of a single subject, are shown in Figure 3. As the threshold increases, the graph density is reduced from ~51% (0 threshold) to ~30% (125 threshold). The level of variation (error bars in Figure 2) is consistent across all thresholds with coefficients of variation from 3.5 to 3.8%. From this point on, the order of all threshold comparisons will be from 0, 25, 50, to 125.

### Node Connectivity

**FIGURE 4:**
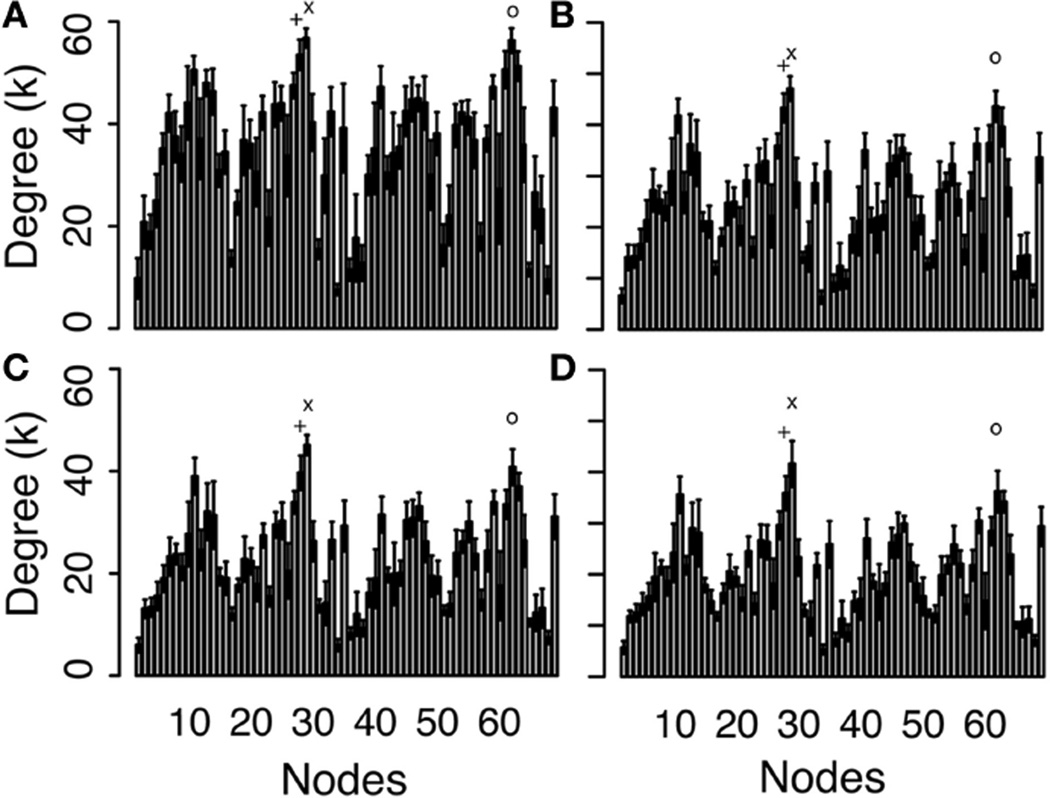
Degree values for each node, averaged across the 10 networks at threshold values of 0 (A), 25 (B), 50 (C), and 125 (D) See Supplemental Data for the list of nodes. The three largest degree values are (x) L superior parietal lobe (Label 28), (o) R superior frontal cortex (Label 62), and (+) L superior frontal cortex (Label 27).

The averages of binary network degree, over all nodes in all networks at each threshold, are shown in Table 1, and Figure 4 shows the average degree value for each node averaged across all 10 networks. These average degree values decrease with increasing threshold, as expected for a binary network, since an increase in the threshold reduces the number of edges (as shown for graph density in Figure 3). The overall shape of the network-averaged node degree values in Figure 4 is maintained at all thresholds (e.g., in particular the highest degree nodes were the same at all thresholds).

**FIGURE 5:**
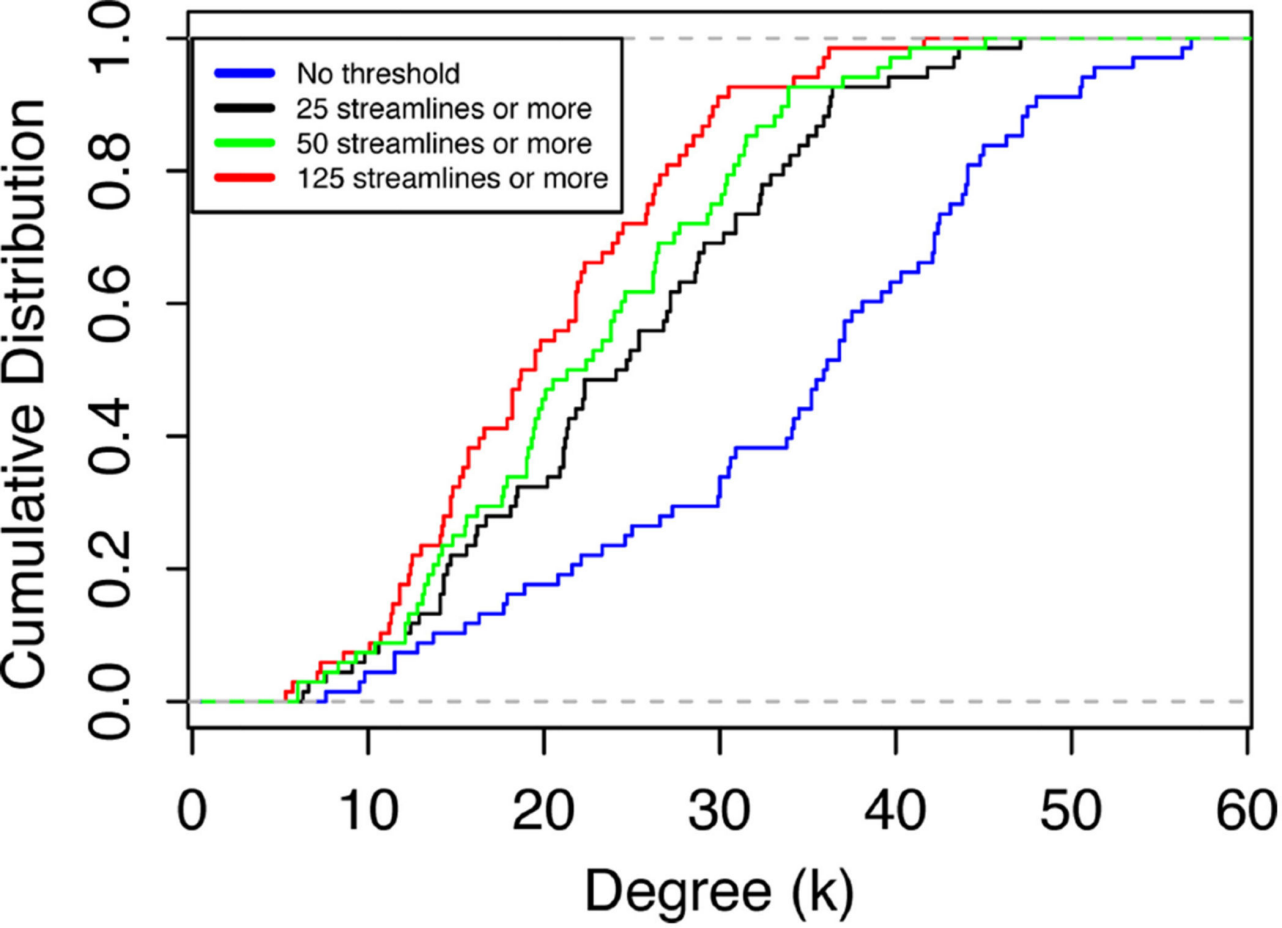
Cumulative degree distribution averages over all nodes in all network at threshold values of 0, 25, 50, and 125 streamlines or more

The nodes with the highest degree are the left (L) superior parietal lobe (Node 28), the right (R) superior frontal cortex (Node 62) and L superior frontal cortex (Node 27). The average node degree of L superior parietal lobe is 56.8, 47.1, 45.1, and 41.6, as the threshold increases, the average degree of R superior frontal cortex is 56.3, 43.6, 40.8, and 36.2, and the average degree of L superior frontal cortex is 53.5, 43.3, 39.7, and 35.9. As the threshold increases, the node degree of paracentral lobules (Node 16 and 50) do not change (this is the only node that did not change degree with threshold changes) and the node degree shows reductions of up to 62% (frontal pole, Nodes 31 and 65). The cumulative degree distributions are shown in Figure 5, showing a shift of the distribution toward lower degree values as the threshold increases.

**FIGURE 6:**
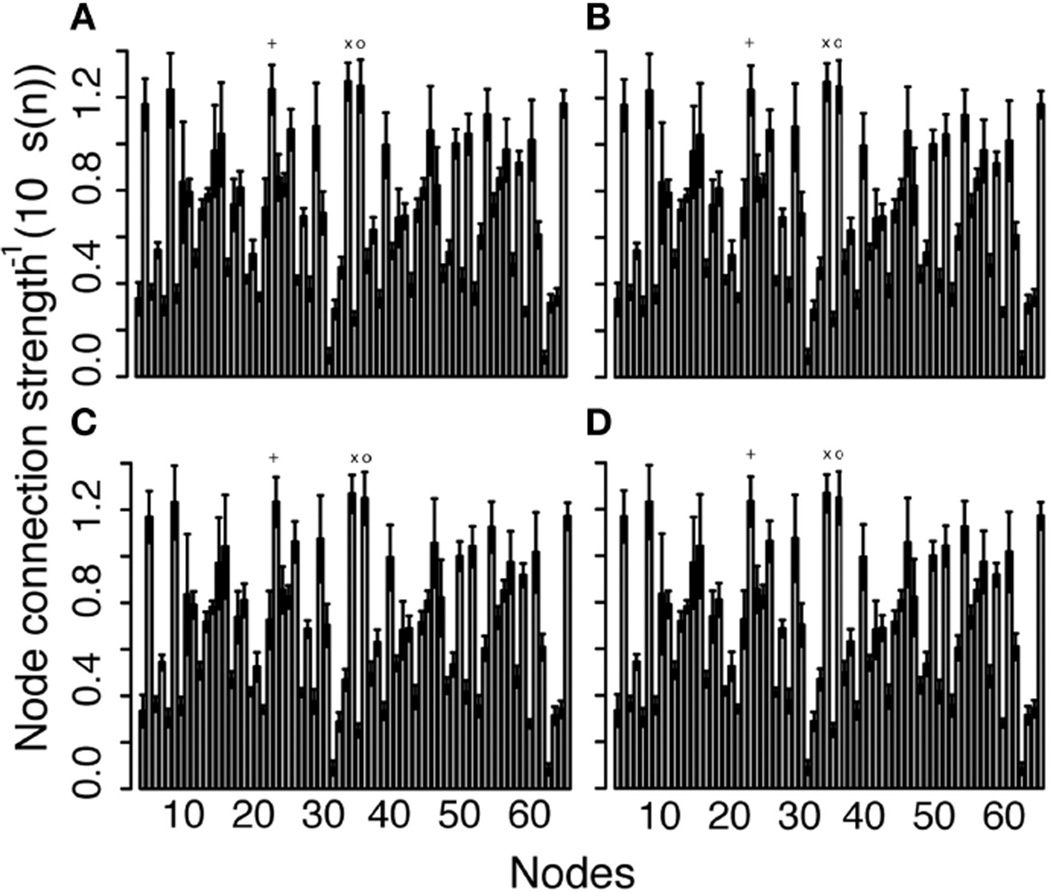
Connection strength of each node averaged across the 10 networks as the threshold increases: (A) threshold 0, (B) 25, (C) 50, and (D) 125 See Supplemental Data for the list of nodes. The three largest node connection strengths are (x) L insula (Node 34), (o) L caudal anterior cingulate (Node 36), and (+) L posterior cingulate (Node 22).

**FIGURE 7:**
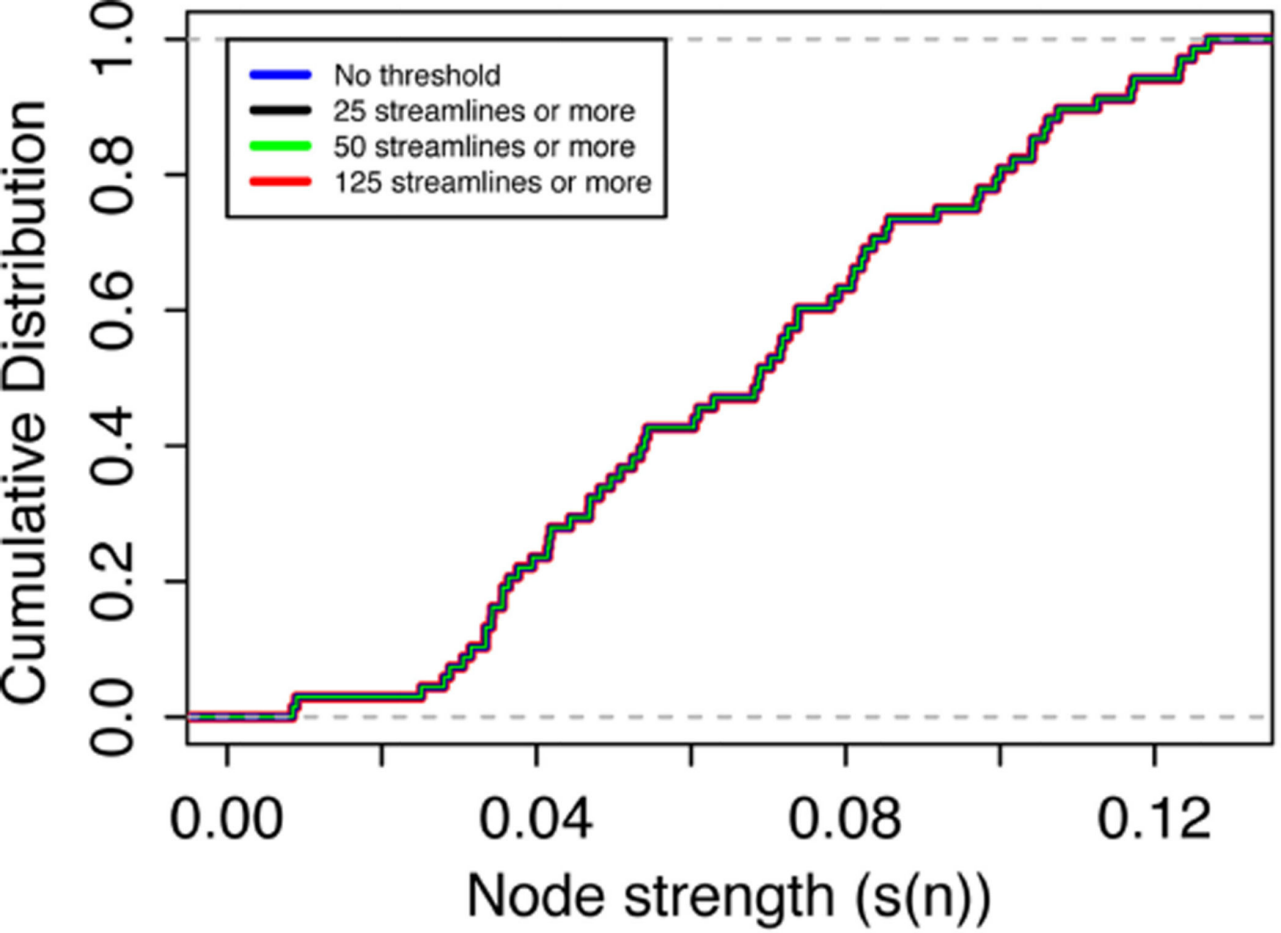
Cumulative node connection strength distribution averaged over all nodes for all networks at threshold values 0, 25, 50, and 125 streamlines or more

The average connection strengths for all nodes over all networks are shown in Table 1 and are relatively constant. As the threshold increases, the connection strength for some nodes do not change and the average node connection strength with the greatest change is only 0.24%, because the edge weight value of the weakest edges are more than 100 orders of magnitude smaller than the majority of nodes. Connection strength for each node averaged over all the networks and variations (Figure 6) did not significantly change as the threshold is varied. This is in stark contrast with the node degree result (Figures 3, 4), which displays clearly visible differences with changes in the threshold. The node strength distribution is almost unchanged and these changes are negligible in as the threshold is changed, as shown in Figure 7. These results indicate that thresholding does not have a significant effect on node connection strength using the edge weight of Equation (15). The nodes with the highest average connection strength at all thresholds are the L fusiform [Node 6, *s(n)* = 0.128], L insula [Node 34, *s(n)* = 0.127], the R caudal anterior cingulate [Node 36, *s(n)* = 0.125] and the L posterior cingulate [Node 22, *s(n)* = 0.123]. These nodes are connected by large coherent WM tracks (represented by large edge weights) that connect these nodes to the rest of brain.

**FIGURE 8:**
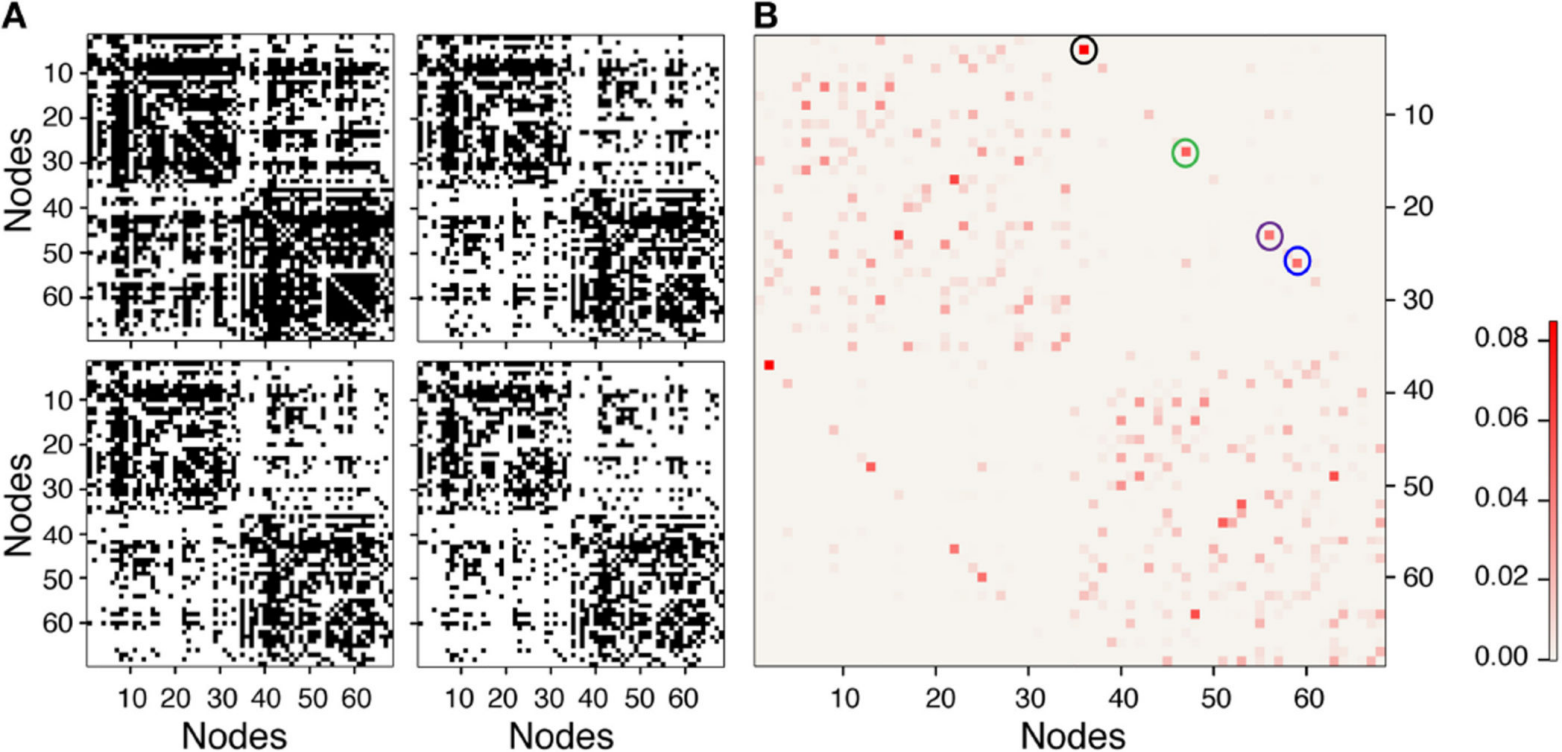
Representative single network binary (A) and weighted (B) adjacency matrices The binary matrices are at thresholds of 0, 25, 50, and 125 from top left to bottom right. Weighted adjacency matrix does not visually change with threshold so only the matrix without threshold is shown in **(B)**. Black, blue, green, and purple circles are the largest edge weights corresponding to interhemispheric connections between left and right caudal anterior cingulate, rostral anterior cingulate, medial orbitofrontal, and posterior cingulate cortices, respectively.

Figure 8 shows a representative single-network, binary adjacency matrix and a weighted adjacency matrix as the threshold is increased. In Figure 8A, the binary adjacency matrix displays significant variation as the threshold increases, while no noticeable difference is observed in the weighted adjacency matrix shown in Figure 8B. Also the most strongly connected nodes stand out in the weighted adjacency matrix. The highest edge weights in the weighted adjacency matrices, averaged over all the networks (shown in the top-right and bottom-left quadrant), represent inter-hemispheric connections, through the corpus callosum, between the L and R caudal anterior cingulate cortex [Nodes 2 and 36 (black circle), *w*(*e*) = 0.0919], the L and R rostral anterior cingulate cortex [Nodes 25 and 59 (blue circle), *w*(*e*) = 0.0564], the L and R medial orbitofrontal cortex [Nodes 13 and 47 (green circle), *w*(*e*) = 0.0553], and L and R posterior cingulate [Nodes 22 and 56 (purple circle), *w(e)* = 0.0508]. All of these edges represent important inter-hemispheric connections that facilitate information transfer from the right to the left side of the brain. In addition, the next six highest edge weights in the adjacency matrix correspond to strong intra-hemispheric connections with values ranging from 0.0353 to 0.0455 (see Supplementary Materials for a complete listing of all edge weights, node degrees and node connection strengths).

### Mean Geodesic Path Length

**FIGURE 9:**
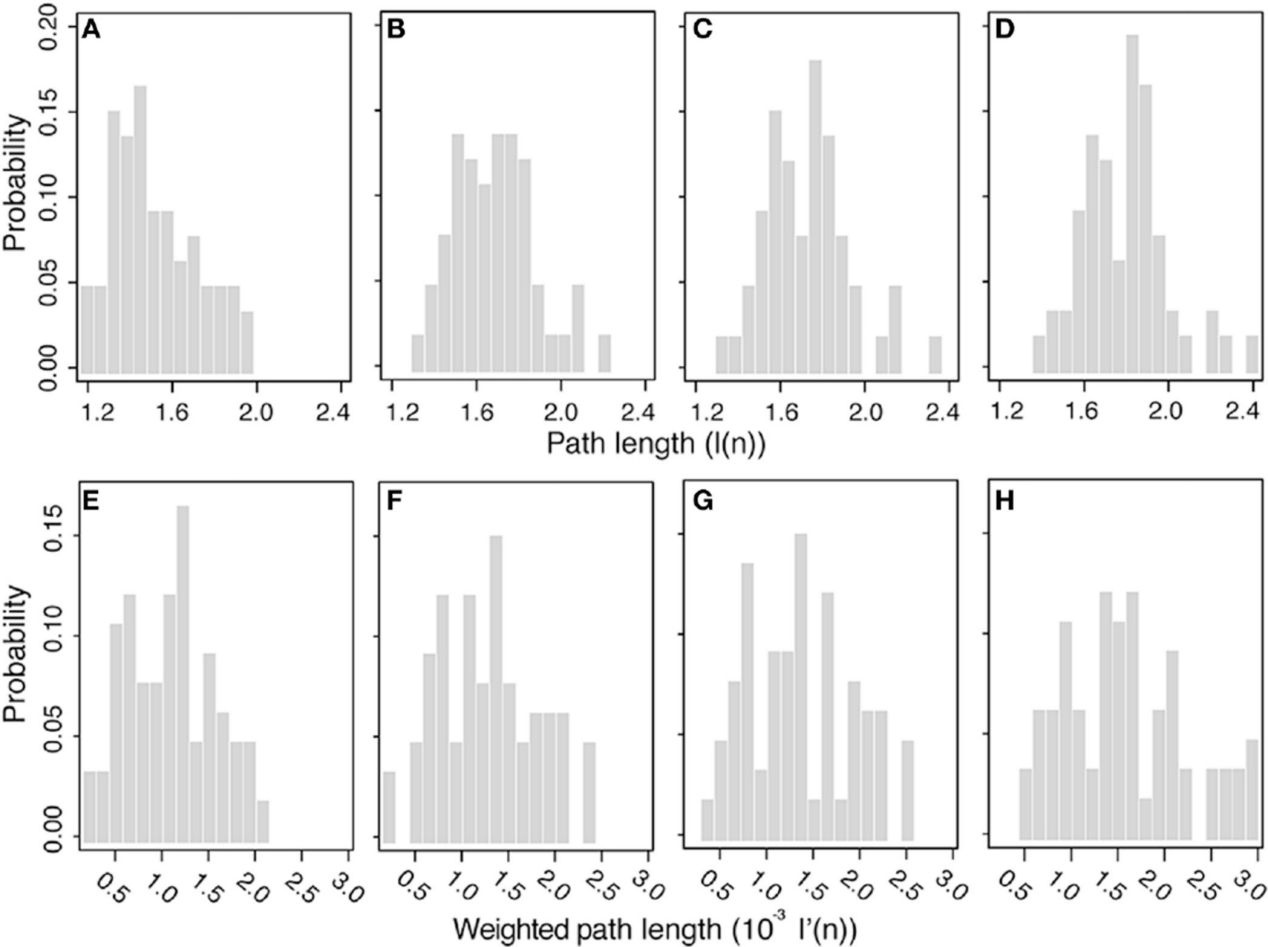
Mean geodesic path length distributions: Binary path lengths at threshold 0 (A), 25 (B), 50 (C), and 125 (D), and weighted path length at threshold 0 (E), 25 (F), 50 (G), and 125 (H)

Figure 9 shows the mean geodesic path length distribution for the binary (Figures 9A–D) and weighted networks (Figures 9E–H) at all thresholds, which suggests that these brain networks are organized to favor shorter path lengths. As thresholds increases, the distribution shifts to longer (binary) and stronger (weighted) path lengths, since shortcuts (“weak” edges) are removed from the network. For all nodes in the binary network at all thresholds, the mean geodesic path length is shorter than 2.5 edges, even at the highest thresholding where the graph density was reduced by 20% (see Figure 3). The mean geodesic path length, for all nodes averaged over the 10 binary networks, is shown in Table 2, and ranges from 1.51 to 1.79 as the threshold increases. L superior parietal lobe (Node 28), which the highest degree values (see Figure 4 and Supplementary Materials), has the shortest mean geodesic path lengths 1.16, 1.31, 1.34, and 1.39, for 0–125 thresholds, respectively. The R superior frontal cortex (Node 62), which has one of the three highest degree values, has the second shortest mean geodesic path lengths of 1.17, 1.36, and 1.41 (0, 25, and 50 thresholds) in networks with high density, while the L lateral occipital lobe (Node 10) displays the second shortest mean geodesic path length value of 1.48 at the lowest density (125 threshold). The average mean geodesic path length over the entire null hypothesis network displayed similar and slightly shorter path lengths, ranging from 1.50 to 1.72, than brain networks, which ranges from 1.51 to 1.79. The short brain path lengths suggest a path of only a few nodes is require to deliver information efficiently in these brain networks, which may suggest a reduction in the probability of information being distorted as it travels through the network.

A broad range of mean geodesic weighted-path lengths were obtained, as shown in Figures 9E–H, with values ranging from 8 × 10^−4^ to 3 × 10^−3^. Removing weak edges did not significantly modify the node strengths (Table 1) or the weighted connectivity matrix (Figure 8), since weak edges only made a small contribution to these values. As threshold changes, the mean geodesic weighted-path length did not share the robustness displayed by the node strength and the weighted connectivity matrix. The mean geodesic weighted-path lengths yields significantly stronger paths as thresholds are increased because weak paths get replaced with stronger paths made up of larger edge weights. Unlike node strength, the weighted path length displays a higher sensitivity to thresholding and shifts the bulk of the distribution to higher values, i.e., to stronger paths, as the thresholds are increased. This effect can also be observed in the average mean geodesic weighted-path lengths obtained at different thresholds (Table 2). The effect of thresholding becomes evident when looking at the strongest paths at each threshold. The strongest path lengths are the R caudal anterior cingulate cortex (Node 36) at high graph densities (0 and 25 threshold), the L caudal anterior cingulate cortex (Node 2) in the 50 threshold network, and the L transverse temporal cortex (Nodes 33 and 67) at low graph density (125 threshold). Table 2 shows that the values of the brain network mean geodesic weighted-path lengths range from 1.08 × 10^−3^ to 1.57 × 10^−3^, while mean geodesic weighted path lengths of the null hypothesis graphs are larger; in the range from 1.40 × 10^−3^ to 2.23 × 10^−3^.

### Clustering Coefficient

**FIGURE 10:**
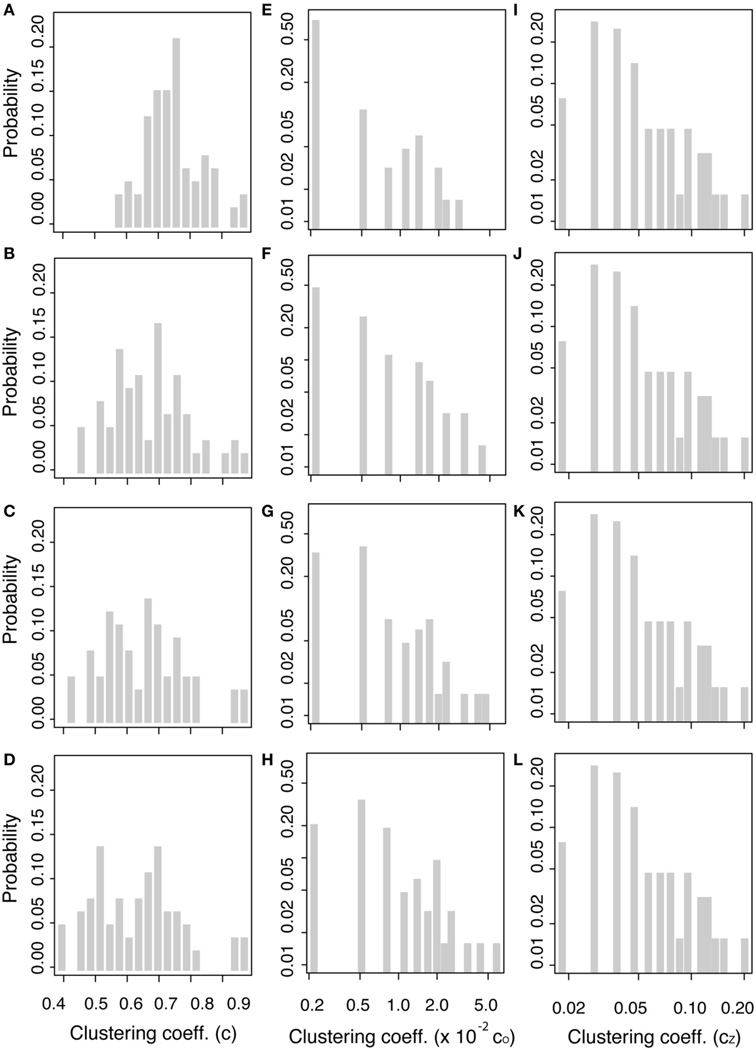
Clustering coefficient distributions Binary clustering coefficient at threshold: 0 **(A)**, 25 **(B)**, 50 **(C)**, and 125 **(D)**. Weighted clustering coefficient, c_*O*_ (see Equation 9),at threshold: 0 **(E)**, 25 **(F)**, 50 **(G)**, and 125 **(H)** and weighted clustering coefficient, c_*z*_ (see Equation 10),at threshold: 0 **(I)**, 25 **(J)**, 50 **(K)**, and 125 **(L)**.

The distribution for clustering coefficients is shown in Figure 10 (in column orientation as threshold increases from top to bottom) for the binary clustering coefficients, c_*i*_, from Equation (8) (Figures 10A–D in linear-linear scale), for the weighted clustering coefficient, *c_O_* from Equation (9) (Figures 10E–H in log-log scale), and for the weighted clustering coefficient, *c_Z_*, from Equation (10) (Figures 10I–L in log-log scale). As the threshold increases, the distribution of the binary clustering coefficient, *c_B_*, shows a high level of clustering, ranging from 0.4 to about 1. The distribution broadens as the threshold increase, which implies that “weak” edges are removed resulting in some nodes losing the strength of the surrounding communities. The nodes with the highest binary clustering coefficient across all thresholds were the L transverse temporal cortex (Node 33) and the L banks of the superior temporal sulcus (Node 1), with mean values all above 0.95 for all thresholds. The distribution of the weighted clustering coefficient, *c_O_*, varies only slightly as the threshold is changed. This variation may be attributed to fact that this clustering coefficient uses node degree in the formula and the streamline count threshold parameter will directly affect the value of the node degree. For the weighted clustering coefficient *c_O_*, the most clustered nodes are the L transverse temporal cortex (Node 33), banks of the superior temporal sulcus (Nodes 1 and 35), and the R transverse temporal cortex (Node 67). At the highest graph density, the banks of the superior temporal sulcus become the third most clustered nodes, while R transverse temporal cortex becomes the second. The distribution for the weighted clustering coefficient, *c_Z_*, does not change visibly as the threshold changes, which is similar to the thresholding stability of the node strength and weighted connectivity matrices. For the clustering coefficient *c_Z_*, the most clustered nodes are the L and R banks of the superior temporal sulcus (Nodes 1 and 35) and the L and R parahippocampal gyrus (Nodes 15 and 49).

Table 2 reports average of the clustering coefficients over the entire network. The average binary clustering coefficient, c_g_, decrease from a value of 0.74–0.63 with increasing threshold, while null hypothesis average clustering coefficient decreased even more from a value of 0.66–0.39 (values not included in Table 2). Average weighted clustering coefficients *c_O_* ranges from 4.42 × 10^−3^ to 10.1 × 10^−3^ and *c_Z_* is 5.25 × 10^−3^ using at all thresholds. The weighted null hypothesis network has significantly smaller weighted clustering coefficients than those in the brain network. The weighted null hypothesis *c_O_* results range from 1.04 × 10^−3^ to 2.81 × 10^−3^, and *c_Z_* has the same value of 1.82 × 10^−3^ at all thresholds. The high level clustering suggests that the brain is not a random network and follows principles of efficient network structures [23]. Since the brain network displays a more clustered organization than the null hypothesis network, this implies that the brain is arranged to allow enhanced communication between communities of nodes (i.e., nodes sharing similar neighbors) and allow brain regions to work together to achieve faster information processing.

The weighted clustering coefficients displayed little variation in their distribution and the average value *c_Z_* did not vary as thresholds were changed, which is similar to the small dependence on threshold of node strength and the weighted connectivity matrix. From these results, one can reason that thresholds are not critical to construct weighted networks; since these network parameters are stable and differences from the null hypothesis are obtained in all graph densities.

### Small Worldness

For both the binary and weighted networks, the ratio of the brain network mean path length to null hypothesis mean path length, λ, and the ratio of the brain network mean clustering coefficient to the null hypothesis mean clustering coefficient, γ, are shown in Table 3. As expected, the binary network displayed γ > 1 and λ ~ 1, which yields a small world parameter, *sw*, with a value > 1 indicating that this binary networks displays the small world property [34]. The weighted approach also displayed γ > 1 and λ ~ 1 yielding *sw* > 1.

## DISCUSSION

This work uses a weighted-network framework to characterize brain topological features, such as small worldness, and compares the results to a binary-network framework. The characterization of brain network properties for both binary and weighted edges are improved when a high number of seeds per voxel are used in dMRI tractography [37, 50]. But employing a large number of seeds per voxel may increase the probability of observing spurious connections [51], which may lead to an anomalous high density of edges in these networks. Also high density networks lead to complications when estimating the small world organization [34], so thresholding is used to eliminate the undesired connections that may affect the network measures. It is unlikely that any particular threshold would completely eliminate all false positive from a constructed network. Therefore, networks with properly weighted edges reduce the effect of false positive connections and the use of thresholds might be avoided. Although this work as focused on the use of thresholding (as known as sparsification) to reduce the number of spurious connections, alternatives methods (pruning) have been proposed in order to better preserve the topological features of the network [52]. These methods might serve as ideal methods for eliminate weak or false positives in connectome studies, but similar network metric comparison results, between binary and weighted networks, would still hold.

**FIGURE 11:**
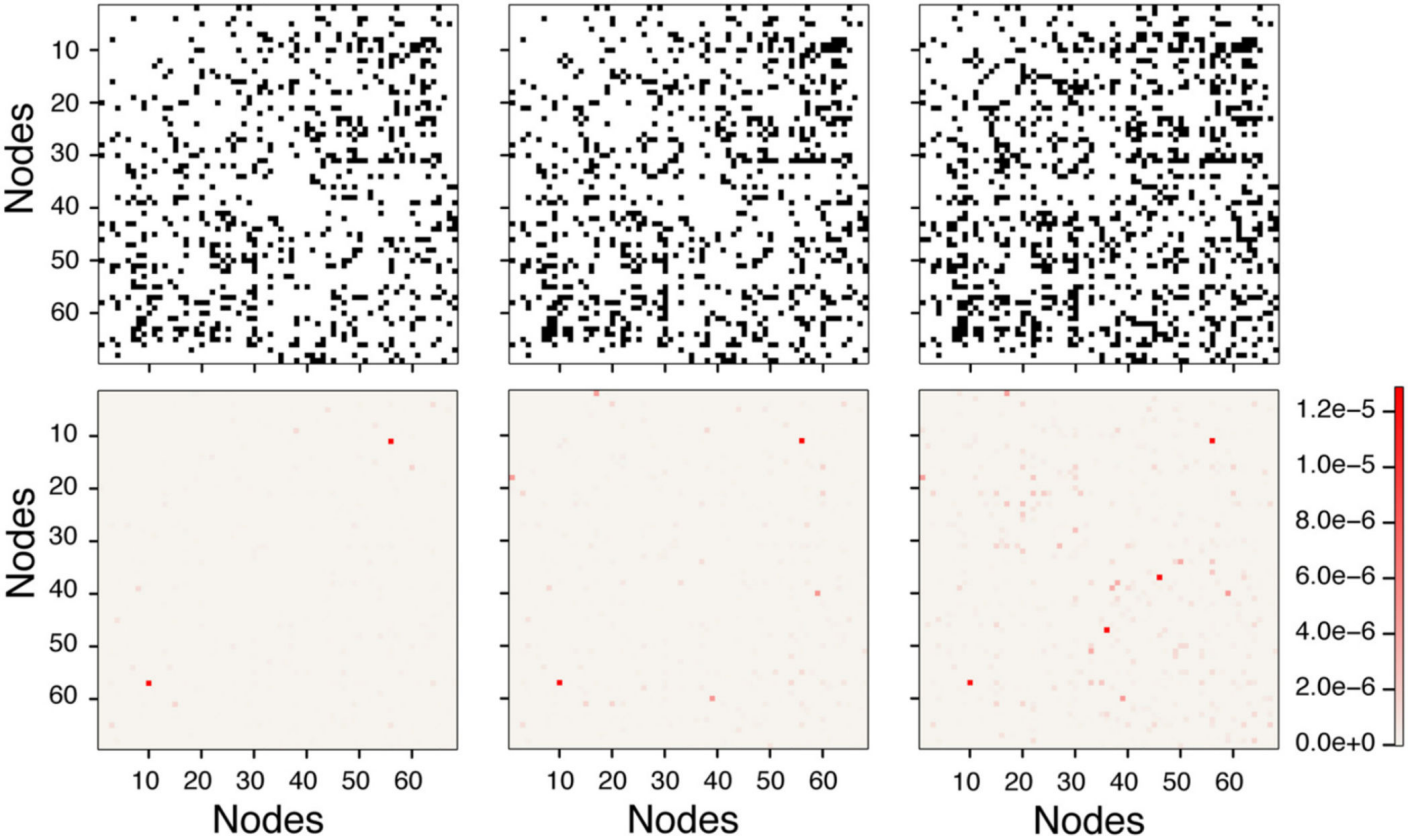
Differences with and without thresholds in binary and weighted adjacency matrices Top row shows the binary matrix changes as edges are removed with increasing threshold. Bottom row shows the associated weighted differences. Left Column shows the difference between the unthresholded (0-threshold) and threshold of 25 streamlines or more (25-threshold). Middle column shows the difference between 0-threshold and 50-threshold. Right column shows difference between 0-threshold and 125-threshold.

As pointed out above, the use of weighted brain network analysis may eliminate the need for thresholding to obtain topological properties in weighted connectomes. In this study as the threshold changes, the variability in connectivity (i.e., degree vs. node strength) across the 10 networks was less in weighted networks (< 10%) than in binary networks (< 15%), as shown in Figures 3, 5. Also removing the edges with a low number of streamlines removes low edge weights (*e*(*w*) ~ 10^−6^), which are small compared to the strong edge weights in the network (*e*(*w*) ~ 10^−2^). Figure 11 shows the differences between binary and weighted adjacency matrices as the threshold changes. The binary adjacency matrix shows a significant number of changes throughout the network. In contrast, the weighted adjacency matrix shows very few changes and the level of the change is negligible. The binary network metrics were highly susceptible to thresholding, as shown in Figures 4, 5, 8A, 9A–D, 10A–D. In contrast, the weighted network metrics displayed far less variation across thresholding, as shown in Figures 6, 7, 10E–L with the exception of the weighted path length (Figures 9E–H). Specific attention is drawn to Figures 5, 7, which show the cumulative distribution of connectivity metrics (node degree and node strength), and the effect of thresholding in the node degree while the node strength have negligible changes. The clustering coefficient introduced by Zhang (Equation 10) displayed a higher resilience to thresholding than the one introduced by Onnela (Equation 9). This might result from Onnela’s normalization by the node degree, *k*, which is a binary measure, while Zhang’s coefficient is an entirely weighted-description of clustering. The binary geodesic path lengths increase as thresholds increase due to the removal of weak edges that serve as shortcuts in dense graphs. In contrast to the other weighted metrics, the geodesic weighted-path length was susceptible to changing thresholds, as shown in Figures 9E–H. Thresholding removes direct “weak” connections between any two nodes as the threshold increases; therefore, weak and short path lengths are replaced with longer, stronger paths. Consequently, the new weighted path length will be significantly stronger than the original “weak” connection. Nonetheless, it still displayed similar weighted path lengths to the null hypothesis networks suggesting effective paths between any two nodes.

Since weak connections are eliminated with thresholding, these connections have not been given attention in brain network studies. But Granovetter argues that weak ties are extremely important in social networks, since it allows for the shorter path lengths observed in social interactions [53]. So the same reasoning might be applied to brain networks, since the brain is organized in a dense mesh that show an intricate web of weak and strong connections, providing a very stable network that is robust to random errors or attacks. Therefore, weak edges may support the integrative capabilities of networks, whereas strong links provide connectivity between neighborhoods [21].

In this work, the small world property of brain networks was successfully determined, in both binary and weighted approaches, as seen in Table 3, even at higher graph densities than estimated for cortical thickness networks [54]. The binary network small worldness parameter increased with an increase in threshold, resulting in a decrease in the density of edges as expected, but small worldness was not observed in the binary dense network without thresholding, where the concentration of edges in the network in high (~50%). With high graph density, the likelihood that the neighbors of any node are connected is high, so the high clustering observed in the non-thresholded binary networks is similar to that in null hypothesis network so small world organization is observed. While binary networks only show sw = 1.53 for a thresholded network of 125, the weighted approach displays sw values larger than 3.5 for all graph densities. Essentially, high graph densities do not conceal the topological features of brain networks in weighted networks. High graph densities of networks obtained from dMRI tractography are certain to include a large number of false positives [51], but the weighting of edges used in this work reduces their relevance in the topological analysis enabling the observation of the topological characteristics of small world networks even at high graph densities.

A limitation of the current tractography approach is that the small edge weights might be true positives while others might be false positives [51]. However, the use of weighted edges reduces the impact of this limitation and allows the generation of connectomes that yield information more relevant to the real small world topology of the brain. Therefore, use of weighted edges loosens the restriction that networks should be sparse, and the need for thresholds, to determine the topological traits of brain networks. In this study, the null hypothesis for each brain network was calculated once per scanning session. This can be a limitation to identify differences in larger populations due to the limitations of sampling in the null hypothesis network. In future studies a larger number of null hypotheses networks should be made for each brain network. Weighted networks displayed small world topology at all thresholds, suggesting that the small world property is an inherent property of the brain network (when properly weighted). The weighted framework preserves properties like small worldness, connection node strength, and clustering coefficient but additional testing needs to be performed with other network measures, like community, cliquishness, rich club, and betweeness centrality, among others. In this study we focused on the small world property and relevant metrics to this topological configuration. The weighted framework presented in this article can be accompanied from additional methods, such as determining the backbone of the weighted connectome [55]. An interesting feature of the weighted backbone procedure of Serrano et al. [55] is the weak edges are not disregarded due to small weights, which is similar to the method used in this study.

The connectome obtained with diffusion MRI and tractography presents a picture at the macroscale where individual streamlines correspond to the coherent pathway generated by the maxima of the probability displacement function at each voxel. This is not intended to be descriptive of single axons, but to represent the cumulative arrangement of axons as white matter, which is quantified as an edge weight. As stated by Sporns [56], “In the case of the brain, it is not necessary to demand that the connectome be an exact replica of the connectional anatomy down to the finest ramifications of neurites and individual synaptic boutons. Instead, the connectome should aim at a description of brain architecture that ranges over multiple levels of organization, reflecting the multiscale nature of brain connectivity.” Hence at the macroscale, the efficiency of brain networks should not rely solely on short path lengths and highest clustering, as implied with binary networks. Efficient brain networks should also include a high density of connections with various weights that enables alternative pathways when disruptions arise in the network.

Ultimately, a complimentary analysis of weighted and binary networks is warranted in topological studies of networks. Strong links provide the backbone of efficient connection in networks and weak links provide a subnetwork that generate a cohesiveness not achieved in sparse networks (similar to the network made up of only strong links) [21]. The results presented here are another example of how to integrate weak links into a real network studies [21] and that the small world topology is descriptive of brain networks regardless of graph density. Methods to estimate the small world parameter have focused on limiting graph density to accurately estimate small worldness [23, 34, 57]. However, real world networks might not inherently be sparse and weak connections will increase the graph density of binary networks to levels that will limit traditional small worldness estimators. In this study, quantifying connection strength enables the estimation of the small world topological property, where the brain connectome is an example of a dense small world network.

## CONCLUSIONS

In previous studies of binary networks in the brain, where all connections are equivalent, the topological structure of the brain network has been shown to display a small world organization [11, 58]. Thresholding is commonly used to mitigate the effects of artefactual connections resulting from tractography, but threshold selection is arbitrary. The results of this study show that thresholding is not necessary for the analysis of network topological organization in a weighted network with the edge weight described in Equation (15). Hence, weighted networks enable the characterization of the brain network topology without resorting to arbitrary thresholding, preserving properties like small worldness, connection node strength, and clustering coefficient. Because of the degree of freedom provided by weighted edges, the weighted connectivity approach described in this paper provides a more accurate representation of connectivity and a more stable framework from which to study brain networks. Using this approach, brain topology can be represented using weak and strong connections that are efficiently arranged to yield a dense and robust small world network.

## Supplementary Material

Data_sheet

## Figures and Tables

**TABLE 1:** Average node degree (*k̂*) values (Equation 4) with associated standard deviation (σ) for binary network and average node connection strength (*Ŝ*) values (Equation 5) and standard deviation (σ) for weighted networks.

	*Binary*		*Weighted*	
		
	*k̂*	σ	*Ŝ* (10^−2^)	σ (10^−3^)
0	36.0	4.49	6.88	7.40
25	24.4	3.49	6.88	7.40
50	21.9	3.14	6.88	7.40
125	19.1	2.81	6.88	7.40

The values were calculated by averaged across all nodes in all 10 networks. The average coefficient of variation is 9.36% for connection strength for all nodes in all networks at all thresholds.

**TABLE 2:** Average binary mean geodesic path length (<*d*>), average mean geometric weighted-path length (<*d^w^*>), and averaged clustering coefficient.

Threshold	Average Mean Geodesic Path length			
	
	Binary		Weighted	
		
	<*d*>	σ	<*d^w^*>(10^−3^)	σ (10^−3^)
0	1.51	0.07	1.08	0.16
25	1.68	0.07	1.28	0.25
50	1.72	0.07	1.37	0.29
125	1.79	0.07	1.57	0.37

Threshold	Clustering coefficient					
	
	Binary			Weighted		
		
	*C_B_*	σ	*C_O_*(10^−3^)	σ(10^−3^)	*c_Z_*(10^−2^)	σ(10^−2^)
0	0.74	0.04	4.42	1.86	5.25	1.24
25	0.67	0.05	7.51	2.71	5.25	1.24
50	0.65	0.06	8.46	2.96	5.25	1.24
125	0.63	0.06	10.1	3.33	5.25	1.25

The values were calculated by averaged across all nodes in all 10 networks and the standard deviation (σ) in the distribution of values is also included.

**TABLE 3:** Weighted and binary network small worldness (*sw*) and associated parameters.

Threshold	γ	σ	λ	σ	*sw*	σ
**BINARY**						

0	1.13	0.07	1.01	0.05	1.12	0.08
25	1.39	0.11	1.02	0.04	1.36	0.12
50	1.47	0.12	1.03	0.04	1.43	0.13
125	1.60	0.14	1.04	0.04	1.53	0.15

**WEIGHTED (*c_O_*)**						

0	4.24	1.79	0.77	0.12	5.51	2.47
25	3.84	1.39	0.78	0.15	4.95	2.04
50	3.68	1.29	0.73	0.16	5.08	2.08
125	3.61	1.18	0.70	0.17	5.13	2.08

**WEIGHTED (*c_Z_*)**						

0	2.89	0.68	0.77	0.12	3.76	1.05
25	2.89	0.68	0.78	0.15	3.72	1.15
50	2.89	0.68	0.73	0.16	3.99	1.27
125	2.89	0.69	0.70	0.17	4.10	1.38

Metrics: γ, see equation 12, λ, see equation 13, sw, see equation 14, and σ is the standard deviation.
